# Functional association of cellular microtubules with viral capsid assembly supports efficient hepatitis B virus replication

**DOI:** 10.1038/s41598-017-11015-4

**Published:** 2017-09-06

**Authors:** Masashi Iwamoto, Dawei Cai, Masaya Sugiyama, Ryosuke Suzuki, Hideki Aizaki, Akihide Ryo, Naoko Ohtani, Yasuhito Tanaka, Masashi Mizokami, Takaji Wakita, Haitao Guo, Koichi Watashi

**Affiliations:** 1 Department of Virology II, National Institute of Infectious Diseases, Tokyo, 162-8640 Japan; 2 Department of Applied Biological Sciences, Tokyo University of Science, Noda, 278-8510 Japan; 3 Department of Microbiology and Immunology, Indiana University School of Medicine, Indianapolis, 46202 USA; 4 Genome Medical Sciences Project, National Center for Global Health and Medicine, Ichikawa, 272-8516 Japan; 5 Department of Microbiology, Yokohama City University School of Medicine, Yokohama, 236-0004 Japan; 6 Department of Virology and Liver Unit, Nagoya City University Graduate School of Medicinal Sciences, Nagoya, 467-8601 Japan; 7 CREST, JST, Saitama, 332-0012 Japan

## Abstract

Viruses exploit host factors and environment for their efficient replication. The virus-host interaction mechanisms for achieving an optimal hepatitis B virus (HBV) replication have been largely unknown. Here, a single cell cloning revealed that HepAD38 cells, a widely-used HBV-inducible cell line, contain cell clones with diverse permissiveness to HBV replication. The HBV permissiveness was impaired upon treatment with microtubule inhibitor nocodazole, which was identified as an HBV replication inhibitor from a pharmacological screening. In the microtubule-disrupted cells, the efficiency of HBV capsid assembly was remarkably decreased without significant change in pre-assembly process. We further found that HBV core interacted with tubulin and co-localized with microtubule-like fibriforms, but this association was abrogated upon microtubule-disassembly agents, resulting in attenuation of capsid formation. Our data thus suggest a significant role of microtubules in the efficient capsid formation during HBV replication. In line with this, a highly HBV permissive cell clone of HepAD38 cells showed a prominent association of core-microtubule and thus a high capacity to support the capsid formation. These findings provide a new aspect of virus-cell interaction for rendering efficient HBV replication.

## Introduction

Hepatitis B virus (HBV) is a member of the *Hepadnaviridae* family, a group of enveloped viruses with carrying approximately 3.2 kb relaxed circular DNA (rcDNA) as their genome^1, 2^. HBV genome encodes four major open reading frames for core, polymerase, surface, and x proteins. Among these, core and polymerase are especially essential for viral DNA replication. Upon the formation of viral covalently closed circular DNA (cccDNA) in the nucleus of an infected hepatocytes, HBV replication is initiated with transcription by using cccDNA as a template to produce viral mRNAs with different length (Fig. S1)^3, 4^. One of the transcripts with approximately 3.5 kb in length, called pre-genomic (pg) RNA, plays an essential role in HBV replication^5^. pgRNA encodes viral polymerase and core proteins. While polymerase interacts with pgRNA, core proteins spontaneously dimerize and then multimerize to assemble into the capsids. The pgRNA-polymerase riboprotein complex is packaged with core proteins to generate nucleocapsids^6^. Inside the nucleocapsids, polymerase reverse-transcribes the pgRNA into complementary minus-stranded DNA and further synthesizes plus-stranded DNA to yield rcDNA, followed by envelopment and virion release (Fig. S1). HBV DNA replication can be evaluated by using cell culture systems including an HBV stable line, HepG2.2.15 cells^7, 8^, and a tetracycline-regulated inducible system, HepAD38 cells^9^, as well as the transient transfection of an HBV-encoding plasmid^10^. It is known that the activity of the HBV replication can be regulated by factors including host cell microenvironment and external stimuli: e.g. HBV replication level is elevated after reaching cell confluent and by treatment with DMSO^8, 11^. However, the molecular basis for determining the permissiveness to HBV replication and the governing virus-host interaction mechanisms remain to be largely clarified.

In this study, we isolated subclones of HepAD38 cells and found that these clones have diversity in the permissiveness to HBV replication. Screening of a pharmacological inhibitor library using a highly HBV-permissive cell clone revealed that microtubules played a significant role in supporting the process for HBV capsid assembly. Moreover, we investigated a relevance of the core-microtubule association in the host permissiveness to HBV replication.

## Results

### Establishment of subclones of HepAD38 and HepG2.2.15 cells with high HBV replication levels

Firstly, we conducted a single cell cloning of HepAD38 and HepG2.2.15 cells, which can induce HBV replication under tetracycline depletion^9^, and permanently replicate HBV^8^, respectively. These cells were seeded on 96 well plates by limiting dilution (see Materials and Methods). At approximately four weeks later, proliferated cell colonies were isolated and expanded in larger plates. Hep38.2-Tet, Hep38.3-Tet, and Hep38.7-Tet cells, as subclones of HepAD38 cells, and HepG2.2.15.7 cells as a subclone of HepG2.2.15 cells grew continuously and could be reproducibly recovered after freezing and thawing among the obtained cell clones.

**Figure 1:**
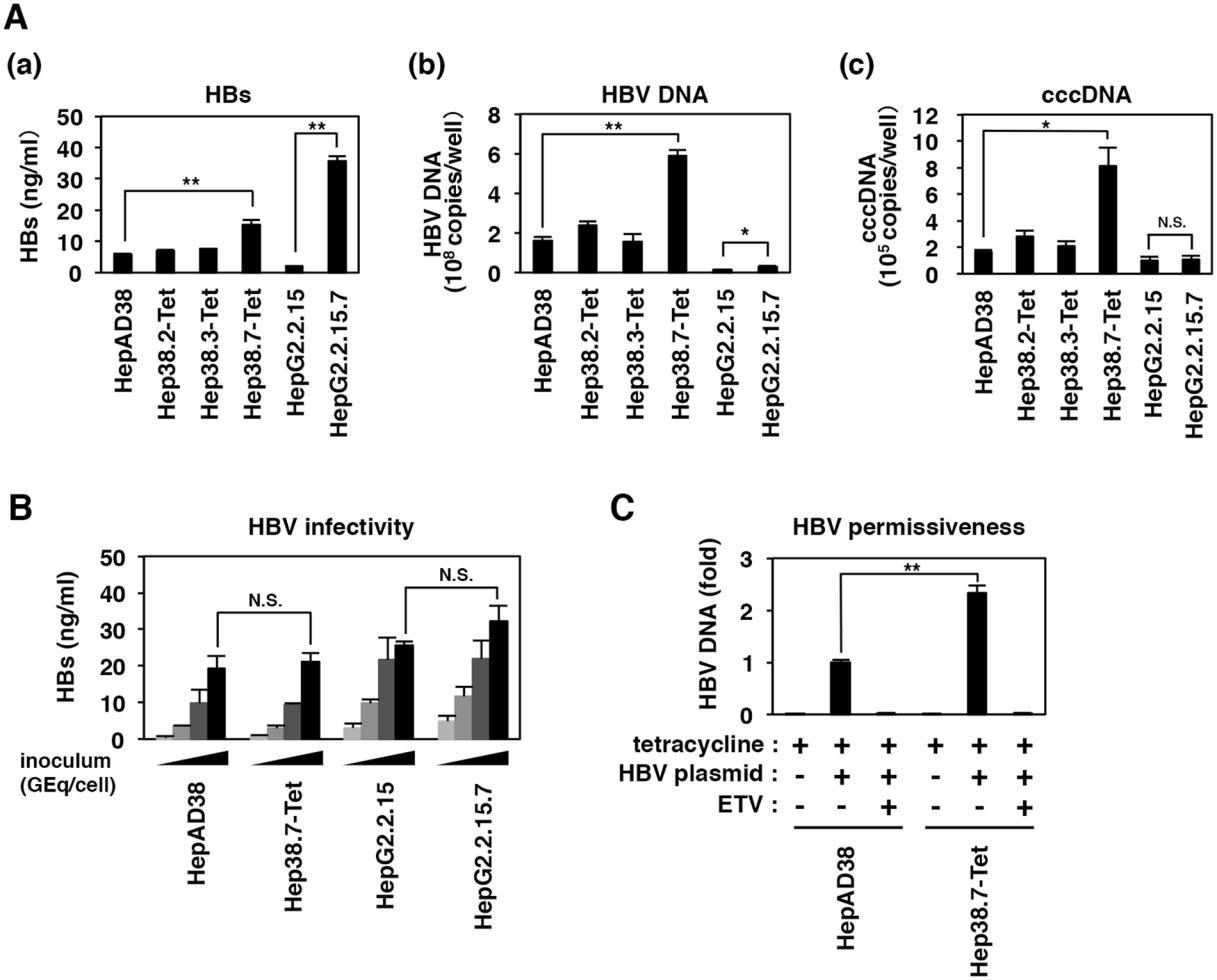
Characterization of novel HBV-replicating cell lines. (**A**) Extracellular HBs (a) as well as intracellular HBV DNA (b) and cccDNA (c) were quantified for each subclone (Hep38.2-Tet, Hep38.3-Tet, Hep38.7-Tet, and HepG2.2.15.7 cells) or the corresponding parental cells (HepAD38 and HepG2.2.15 cells) at 9 days post-seeding (the cells reached confluent at 3 days after seeding, when tetracycline was depleted from HepAD38, Hep38.2-Tet, Hep38.3-Tet, and Hep38.7-Tet cells, and continued for culturing for further 6 days). Hep38.7-Tet cells showed the highest HBV DNA and cccDNA level among the cell clones. (**B**) HBV in the culture supernatant of HepAD38, Hep38.7-Tet, HepG2.2.15 and HepG2.2.15.7 cells was inoculated to HepaRG cells with different amount of HBV inoculum (667, 2,000, 6,000, 18,000 GEq/cell) to examine the infectivity by monitoring HBs secreted from HepaRG cells at 12 days post-infection. (**C**) HepAD38 and Hep38.7-Tet cells were transfected with empty vector or an expression plasmid for HBV. After transfection, these cells were treated with or without 1 μM entecavir (ETV) for 72 h in the presence of tetracycline. Nucleocapsid-associated HBV DNA was quantified by real time PCR. Statistical significance was determined by using Student’s t test (*P < 0.05, **P < 0.01, N.S.; not significant).

Next, we quantified HBV surface proteins (HBs) produced into the culture supernatant and intracellular HBV DNA and cccDNA for the above subclones as follows: After seeding the cells and letting them reached confluent at three days post-seeding, we induced HBV replication in these cells by culturing for six days in the absence of tetracycline and then recovered the culture supernatant to quantify HBs and the cells to detect HBV DNA and cccDNA. As shown in Fig. 1A, while Hep38.2-Tet and Hep38.3-Tet cells produced the equivalent levels of HBs to the parental HepAD38 cells, Hep38.7-Tet cells produced approximately 3 times higher amount of HBs than HepAD38 cells (Fig. 1A-a). HBV DNA and cccDNA in Hep38.7-Tet cells were 3–5 times higher than those in the parental HepAD38 cells, while Hep38.2-Tet and Hep38.3-Tet clones exhibited similar level with HepAD38 cells (Fig. 1A-b,c). Such result has also been seen previously by Southern blot^12^. These data suggest that HBV replicates more efficiently in Hep38.7-Tet cells than in its parental cells or other cell clones. However, sequence analysis indicated no nucleotide substitution in HBV DNA from Hep38.7-Tet cells from that from HepAD38 cells. Furthermore, HBV virions produced from Hep38.7-Tet cells showed similar infectivity to that from the parental HepAD38 cells, examined in the HepaRG cell infection assay with viral inoculum being normalizing by HBV DNA copy genome equivalent (Fig. 1B). Moreover, under non-HBV induction condition, Hep38.7-Tet cells produced nucleocapsid-associated HBV DNA approximately 2.3 times more than HepAD38 cells upon transfection with an HBV-encoding plasmid, carrying 1.24 times length of full length HBV genome (pHBV1.24)^13^ (Fig. 1C). HBV RNA level as well as the transfection efficiency were equal between these two cell lines (Fig. S2A,B). These data suggest that Hep38.7-Tet cells have a more preferable cellular environment for efficient HBV replication especially for a post-transcriptional process (This will be addressed later).

On the other hand, HepG2.2.15.7 cells produced approximately 18 times higher HBs than its parental HepG2.2.15 cells (Fig. 1A-a), while HBV DNA and cccDNA levels in this cell clone were only 1–2 fold higher compared with those in HepG2.2.15 cells (Fig. 1A-b,c). HBV DNA sequences from HepG2.2.15.7 and HepG2.2.15 cells were 100% identical, and the infectivity of progeny virions were consistently equivalent (Fig. 1B). These observations infer a possibility that the host condition of HepG2.2.15.7 cells was more capable of producing HBs.

### Nocodazole reduced HBV permissiveness

Making use of Hep38.7-Tet cell line, we screened a library consisting of cell targeting-pharmacological agents to identify host factors affecting HBV replication. After Hep38.7-Tet cells reached confluent to inactivate cell cycle and achieve efficient HBV replication^8^, tetracycline was removed to induce HBV replication and the cells were treated with library compounds for six days. The culture supernatant and the cells were harvested to quantify HBV DNA and cell viability, respectively. Compounds decreasing the supernatant HBV DNA to less than 20% without reducing cell viability less than 80% were selected as hits. Among the obtained hits, nocodazole exhibited as the most potent inhibitor of HBV replication.

**Figure 2:**
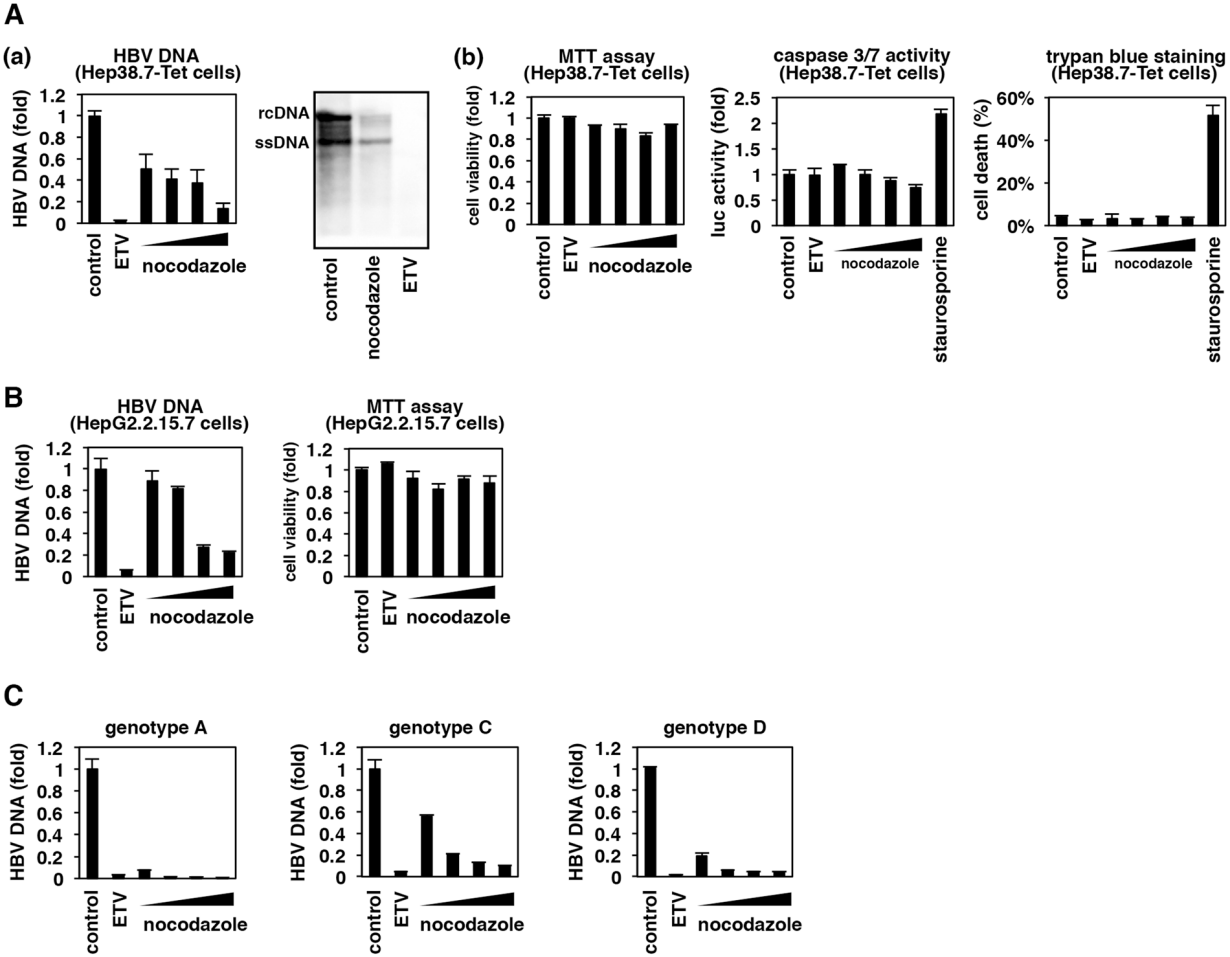
HBV replication was reduced upon nocodazole treatment. Hep38.7-Tet (**A**) and HepG2.2.15.7 (**B**) cells were treated with 1 μM ETV, 0.5, 1, 2.5 and 5 μM nocodazole, or left untreated (“control”) for 6 days. 1 μM staurosporin used as a positive control for exhibiting cell apoptosis were treated to the cells for 24 h. Intracellular HBV DNA (**A**-a left, B left) were quantified by real time PCR. Nucleocapsid-associated HBV DNA upon treatment with or without compounds for 9 days was also detected by Southern blot (**A**-a right). MTT assay (**A**-b left, B right), caspase3/7 assay (**A**-b center), and trypan blue staining (**A**-b right) were also shown to quantify cell viability or apoptotic cell death. (**C**) HepG2 cells transfected with a plasmid encoding different HBV genotypes (A, C, or D) were treated with or without 1 μM ETV or 1, 2.5, 5 and 10 μM nocodazole for 72 h. HBV replication was examined by quantifying nucleocapsid-associated HBV DNA.

Nocodazole was known to disrupt cellular microtubules and arrest cell cycle^14^. However, in this study, we performed all the compound treatment experiments with non-dividing cells under confluent condition, and nocodazole did not show any significant cytotoxicity by MTT assay (Fig. 2A-b, left and B, right). This observation was further confirmed by caspase assay and trypan blue staining (Fig. 2A-b center, right and S3A). Under this condition, nocodazole drastically decreased HBV replication level in Hep38.7-Tet cells in a dose-dependent manner, as shown by intracellular HBV DNA qPCR (Fig. 2A, left) and Southern blot of nucleocapsid-associated HBV DNA (Fig. 2A, right).

Similar observations were obtained in HepG2.2.15.7 cells (Fig. 2B, left), suggesting that the nocodazole effect was not due to an artificial inactivation of tetracycline-regulated promoter of Hep38.7-Tet cells. Moreover, nocodazole reduced the replication of different HBV genotypes in virus genome transfected HepG2 cells, including A, C, and D without significant cytotoxicity (Figs 2C and S3B). The above results clearly indicated that cellular permissiveness to HBV replication was impaired upon nocodazole treatment.

### Incapable capsid formation in nocodazole-treated cells

**Figure 3:**
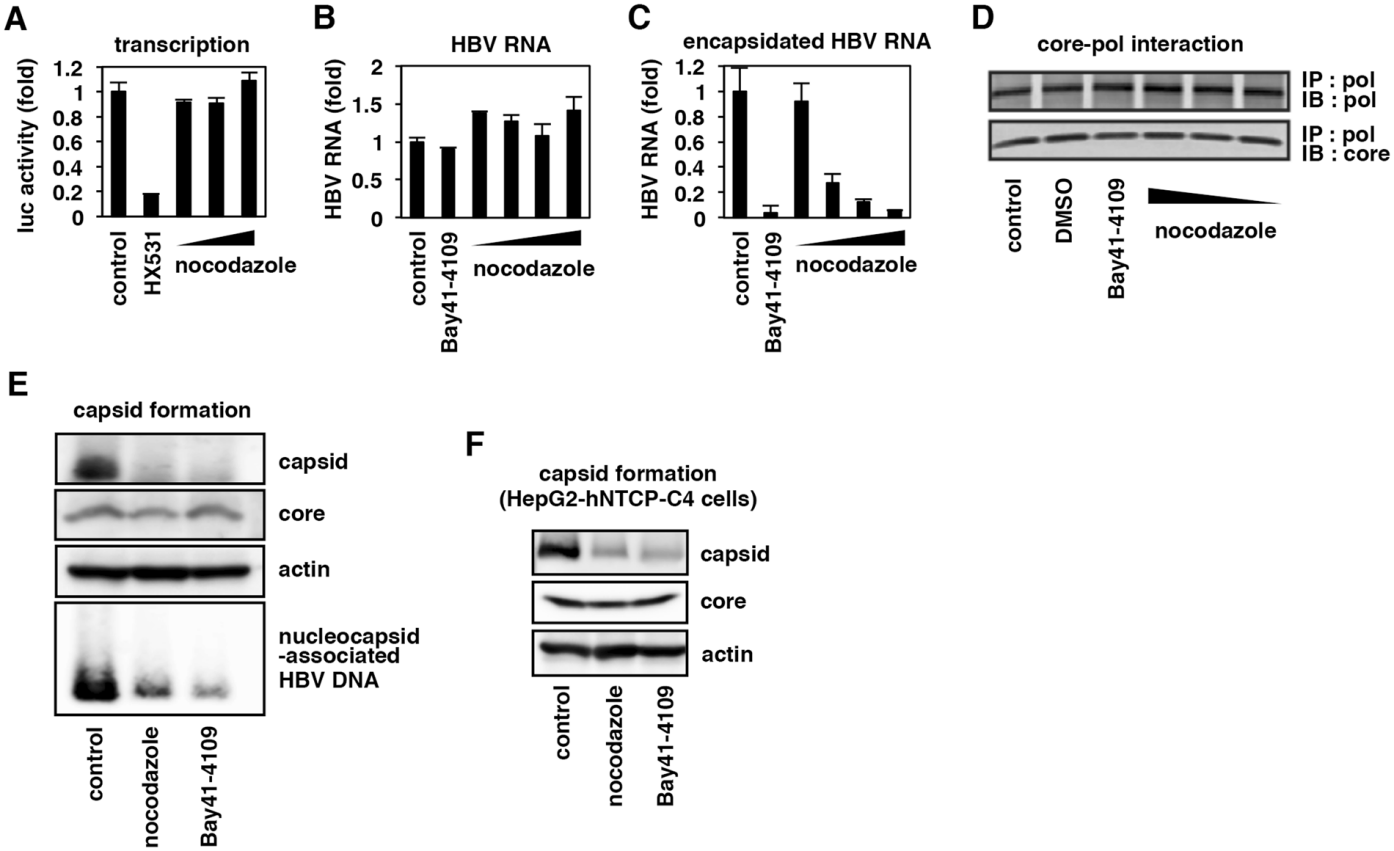
HBV capsid formation was attenuated in the cells treated with nocodazole. (**A**) HepG2 cells transfected with a reporter plasmid carrying the HBV core promoter upstream of the luciferase gene were treated with 10, 20 and 40 μM nocodazole or 30 μM HX531 as a positive control for 7 h, and luciferase activity driven by the HBV core promoter was measured. (**B**,**C**) HepG2 cells transfected with an HBV-encoding plasmid were treated with or without 1 μM Bay41–4109 or 1, 2.5, 5 and 10 μM nocodazole, and then total HBV RNA (**B**) and encapsidated HBV RNA (**C**) were quantified by real time RT-PCR. (**B**) shows relative value of HBV RNA normalized by GAPDH mRNA. (**D**) HepG2.2.15.7 cells treated with or without 0.1% DMSO, 1 μM Bay41-4109 or 1, 5 and 10 μM nocodazole for 6 days were harvested and then pulled down with anti-polymerase antibody to detect HBV polymerase (upper) and core (lower). (**E**) HepG2.2.15.7 cells were treated with 10 μM nocodazole or 1 μM Bay41-4109 for 6 days or left untreated (control). Capsid and nucleocapsid-associated HBV DNA in these cells were detected with a native agarose gel electrophoresis followed by immunoblot and Southern blot, respectively. Total HBV core and actin proteins were also determined by immunoblot. (**F**) Capsid formation (upper) as well as total core (middle) and actin protein as an internal control (lower) were detected in HBV-infected HepG2-hNTCP-C4 cells upon treatment with or without 10 μM nocodazole or 1 μM Bay41-4109. Capsid formation was impaired upon nocodazole treatment.

We investigated the activity of each major steps in the HBV life cycle upon nocodazole treatment (Fig. S1). Firstly, the transcription activity of HBV core promoter, evaluated by a reporter assay using a plasmid carrying luciferase reporter gene controlled by the core promoter, was not affected by nocodazole, in contrast to a remarkable repression of HBV transcription by HX531, a retinoid X receptor antagonist, served as a positive control^15^ (Fig. 3A). Next, we found that the production of total HBV RNA in HepG2 cells transiently transfected with an HBV-encoding plasmid pHBV1.24 was not impaired by nocodazole (Fig. 3B). However, the encapsidation of HBV RNA was dramatically blocked in nocodazole-treated cells dose dependently, which was similar to the treatment with Bay41-4109, a known HBV core assembly inhibitor^16^ (Fig. 3C). Furthermore, the interaction between HBV core and polymerase required for HBV RNA encapsidation, which was examined as described previously^17^, was not apparently affected by nocodazole or Bay41-4109 (Fig. 3D). Interestingly, intracellular HBV capsid formation examined on a native agarose gel was greatly reduced by treatment with nocodazole or Bay41-4109, and this was correlated with a reduction in nucleocapsid-associated HBV DNA, without affecting total core protein level itself (Fig. 3E). Similar observation was obtained in a more physiologically relevant HBV-infected cells that support the whole HBV life cycle^18^, which showed the reduction in capsid formation by treatment with nocodazole and Bay41-4109 (Fig. 3F). The above data clearly suggest that the observed low HBV replication in nocodazole-treated cells was due to the incapability of capsid formation.

### Inefficient capsid formation and core-tubulin interaction by microtubule disassembly

**Figure 4:**
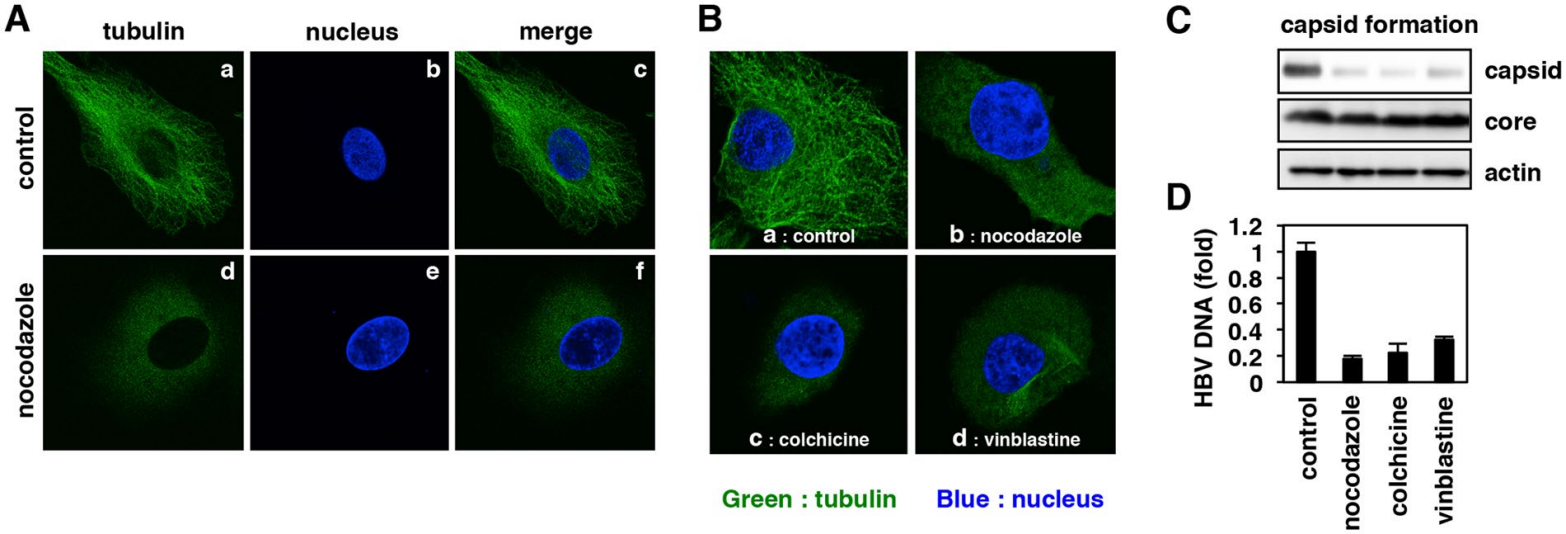
Inefficient capsid formation upon microtubule disruption. (**A**,**B**) Tubulin (green) and the nucleus (blue) were observed in HepG2 cells treated with or without nocodazole, colchicine and vinblastine for 24 h. Merged patterns of green and blue signals are indicated in (**A**) panels c and f and (**B**). (**C**) Capsid formation as well as total core and actin proteins in HepG2.2.15.7 cells treated with or without nocodazole, colchicine, vinblastine and Bay41-4109 were detected as shown in Fig. 3E. (**D**) HBV DNA was quantified in HepG2.2.15.7 cells treated with ETV, nocodazole, colchicine and vinblastine for 6 days. Incapable capsid formation in microtubule-disrupted cells.

It has been reported that core assembly could be readily observed *in vitro* by mixing the recombinant core protein consisting of the assembly domain (1-149 aa) and salt^19–22^. However, it remains elusive what host conditions regulate the capsid formation in cellular settings. Nocodazole is known to depolymerize and destabilize the microtubules in the cells^23^. As shown in Fig. 4A, microtubules-like fibriforms were clearly observed by staining tubulin in untreated HepG2 cells, but this network was disrupted and the tubulin was diffusely distributed in the cells treated with nocodazole (Fig. 4A, green). To examine whether the microtubules were functionally associated with capsid formation, we treated HepG2 cells with other microtubule disassembly agents, specifically colchicine and vinblastine. Similar to nocodazole, treatment with cholchicine and vinblastine disrupted the microtubules-like fibriforms (Fig. 4B, panels b–d), and markedly impaired the capsid formation of HBV and the subsequent DNA replication without affecting the total core protein level and cell viability (Fig. 4C and D).

**Figure 5:**
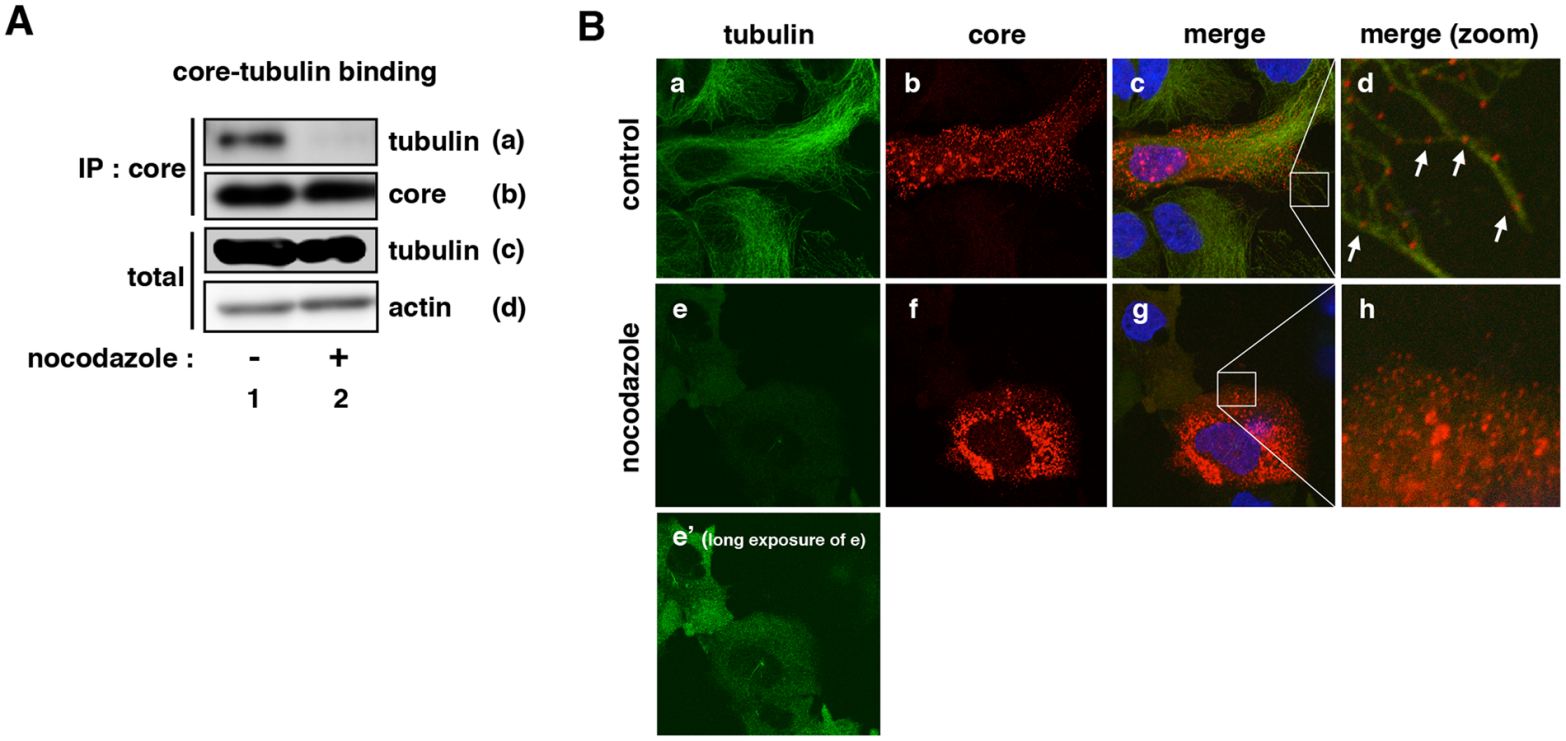
Interaction between tubulin and core protein was interrupted upon nocodazole treatment. (**A**) HepG2 cells transiently overexpressing FLAG-tagged HBV core protein (FLAG-core) were cultured in the presence or absence of nocodazole, and harvested to immunoprecipitate with anti-core antibody and to detect tubulin (a) and core (b) in the precipitates. Tubulin (c) and actin as an internal control (d) were also detected in the total cell lysate without immunoprecipitation. (**B**) HepG2 cells transiently transfected with an HBV-core expression plasmid were treated with nocodazole, and then were stained for HBV core (red), tubulin (green) and the nucleus (blue) by immunofluorescence analysis. Panels d and h are the zoomed in patterns of the inset shown in c (merged panels of a and b) and g (merged panels of e and f), respectively. The exposure time of the figures for “control” (a–d) and “nocodazole” (e–h) is the same. Panel e’ shows a picture of panel e with longer exposure time. Microtubule disassembly induced the dissociation of core-tubulin interaction.

Microtubules are cell cytoskeleton consisting of α,β-tubulin and are generally involved in the transport of cellular molecules and components including mRNA, protein and vesicles. It has been reported that the microtubules are involved in supporting the replication of various viruses including hepatitis C virus and influenza virus^24–27^. Interestingly, it was reported that the microtubules are required for HBV entry before the nuclear translocation and cccDNA formation (Fig. S1)^28^, however, no report has shown the role of the microtubules in HBV replication. As an observation for the reduced HBV replication in microtubule-disrupted cells, we found the dissociation of core-tubulin interaction. In HepG2 cells, core protein was co-localized preferentially with tubulin-positive fibriforms (Fig. 5B, panels a–d) and was able to co-precipitate with tubulin by immunoprecipitation assay (Fig. 5A, lane 1). In contrast, when the fibriforms were disrupted by nocodazole, tubulin-core association disappeared (Fig. 5A, lane 2), and the core localization was changed to the perinuclear region (Fig. 5B, panels e–h). Thus, maintaining microtubules structures is likely to be important for the core-tubulin interaction and efficient capsid formation.

### High core-tubulin association and the efficient capsid formation in Hep38.7-Tet cells

**Figure 6:**
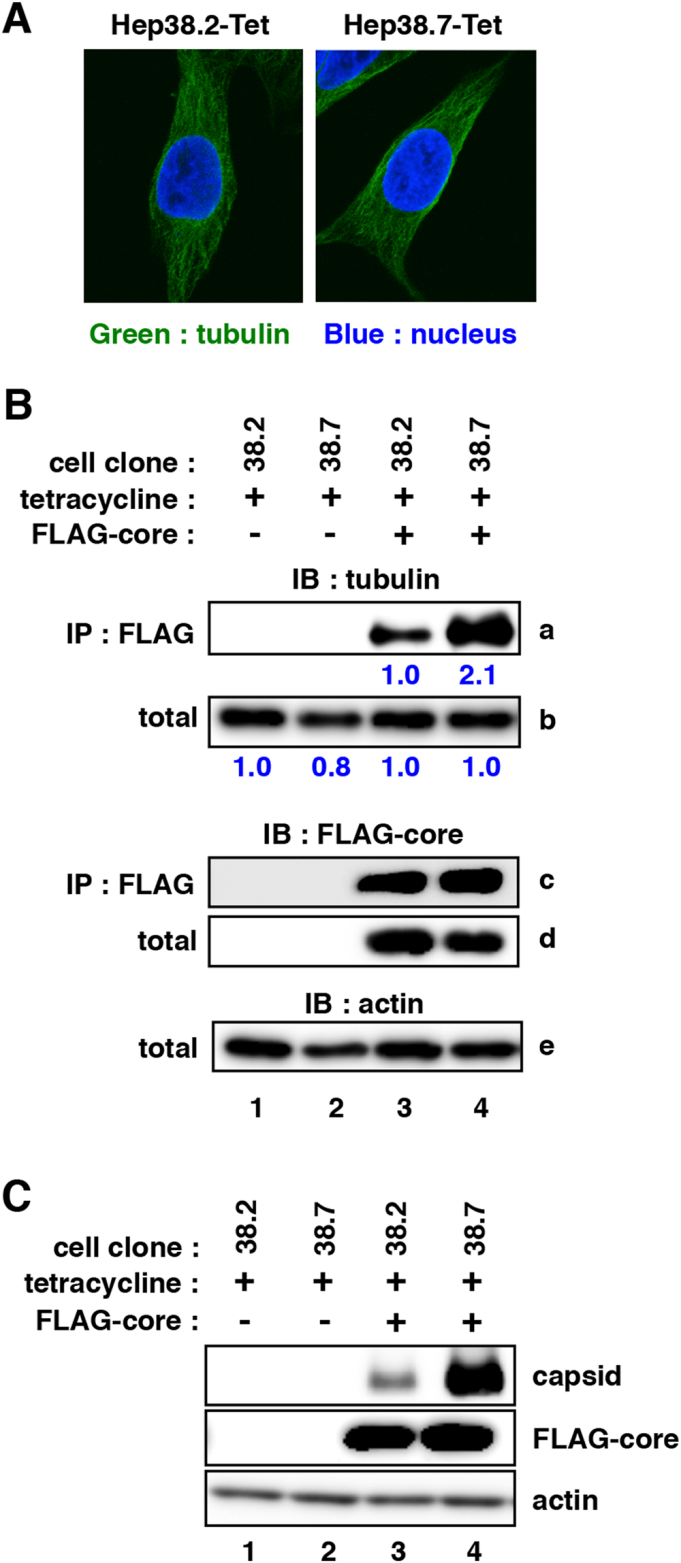
Interaction of core and tubulin in Hep38.7-Tet cells and Hep38.2-Tet cells. (**A**) Tubulin (green) in Hep38.7-Tet and Hep38.2-Tet cells were observed as shown in Fig. 4B. (**B**) Hep38.7-Tet (lanes 2 and 4) and Hep38.2-Tet (lanes 1 and 3) cells were transfected with an expression plasmid for FLAG-core (lanes 3 and 4) or the empty vector (lanes 1 and 2) for 48 h in the presence of tetracycline. These cell lysates were immunoprecipitated with an anti-FLAG antibody (panels a and c) or were recovered without immunoprecipitation (“total”: panels b, d, and e). Tubulin (panels a and b), FLAG-core (panels c and d) and actin (panel e) were detected by immunoblot. Expression levels for tubulin in panels a and b are quantified by densitometry and are shown as relative levels in blue characters below the panels. (**C**) Capsid formation (upper) as well as the expression for FLAG-core (middle) and actin (lower) in the cells prepared as in (**B**) were detected.

As shown in Fig. 1, a single cell cloning provided a variety of cell clones supporting different levels of HBV replication. We characterized two cell clones, Hep38.7-Tet and Hep38.2-Tet cells, which showed relatively high and low permissiveness to HBV replication, respectively (Fig. 1). The microtubules of these cells showed an apparent similar pattern when observed with an anti-α-tubulin antibody by fluorescent microscopy (Fig. 6A). However, we found a remarkable difference in the interaction between core and tubulin. FLAG-tagged HBV core was co-precipitated with tubulin both in Hep38.7-Tet and Hep38.2-Tet cells, but the interaction level was remarkably higher in Hep38.7-Tet cells compared to that in Hep38.2-Tet cells (Fig. 6B panel a) at the similar transfection efficiency between these two cell lines (Fig. S4). This high core-tubulin association resulted in the efficient capsid formation in Hep38.7-Tet cells (Fig. 6C). Thus, the strong microtubule-core interaction in Hep38.7-Tet cells may contribute to its high permissiveness to HBV replication, though the underlying mechanism of cell clone-specific microtubule-core interaction remains obscure.

## Discussion

In the present study, we established and characterized new cell clones from HepG2.2.15 and HepAD38 cells. While the levels for HBV DNA and cccDNA in HepG2.2.15.7 cells were only 1–2 fold higher, HBs showed approximately as much as 18 times higher than that in HepG2.2.15 cells (Fig. 1). Given that HBV DNA sequence is identical among cells, HepG2.2.15.7 cells possibly acquire a host condition to augment S-RNA transcription, HBs translation, secretion, or stability. These steps are actually reported to be regulated by host factors including nuclear factor 1 (HNF-1), CCAAT binding factor (CBF), Rab7, Vps4 and the endosomal sorting complex required for transport (ESCRT) III^29–33^. In any reason, HepG2.2.15.7 cells are useful for efficiently analyzing the mechanisms for HBs or subviral particle metabolism. On the other hand, we also established Hep38.7-Tet cells showing higher permissiveness to HBV replication compared with the parental HepAD38 or its sister cell clones (Fig. 1). Using Hep38.7-Tet cells, we identified that nocodazole reduced HBV replication levels (Fig. 2). Nocodazole depolymerizes cellular microtubules as well as arrests cell cycle that can lead to apoptosis, which although totally depends on cell condition, especially density^34^. In the present study under cell confluent condition and no apparent cell death observed by nocodazole, microtubule depolymerization dissociated core-tubulin interaction and blocked capsid assembly. These results suggest the critical role of microtubules in capsid assembly during HBV replication. Interestingly, this core-tubulin interaction was prominent in Hep38.7-Tet cells (Fig. 6B and C), which suggest that the core-tubulin interaction machinery may determine high HBV replication permissiveness of Hep38.7-Tet cells. It is also interesting to know whether the role of microtubule in HBV replication is conserved in other cell types or *in vivo* settings, which is a future subject to be analyzed in detail by using a mice model.

Thus far, the roles of microtubules in the replication of different viruses have been reported, including working for transporting viral components and as a site of replication: Vesicular stomatitis virus utilized microtubules as a transport pathway for migration of viral nucleocapsids to the plasma membrane for virus assembly^35^. During influenza A virus replication, viral ribonucleoproteins translocate from the nucleus to the pericentriolar recycling endosome through rab11 and microtubule dependent manner^25^. Microtubules-associated septin 9 and PtdIns5P support the formation of lipid droplets to efficiently assemble HCV particles^24^. Dengue virus 2 possibly assembles on microtubules utilized as a scaffold through interaction of microtubules with viral E protein^36^. Our results showing the functional association of the cellular microtubules with HBV capsid formation raised at least two possibilities: 1) Microtubules function to transport the components of HBV capsid to the field of capsid assembly to facilitate HBV replication; 2) Capsid formation occurs on or in association with the microtubules. Further in depth analysis will provide significant information on the mechanisms of microtubule involvement in viral assembly.

In conclusion, by using chemical probes, we identified that the host microtubules play a significant role in supporting HBV capsid formation. This mechanism can be one of the determinants of host permissiveness to HBV replication. Our study presents a new aspect for understanding the HBV-host interaction for achieving an optimal viral replication in host cells, and for developing host targeting agents as novel HBV therapeutics.

## Materials and Methods

### Cell culture

HepG2, HepAD38 (kindly provided by Dr. Christoph Seeger at Fox Chase Cancer Center)^9^, HepG2.2.15^8^, Hep38.2-Tet, Hep38.3-Tet, Hep38.7-Tet, and HepG2.2.15.7 cells were cultured with DMEM/F-12 + GlutaMax (Invitrogen) supplemented with 10 mM HEPES (Invitrogen), 200 unit/ml penicillin, 200 μg/ml streptomycin, 10% FBS and 5 μg/ml insulin in the presence (HepAD38, Hep38.2-Tet, Hep38.3-Tet, Hep38.7-Tet, HepG2.2.15 and HepG2.2.15.7) or absence (HepG2) of 400 μg/ml G418 (Nacalai). HepAD38, Hep38.2-Tet, Hep38.3-Tet, and Hep38.7-Tet cells were cultured with 0.4 μg/ml tetracycline during maintenance and passage, and were cultured in the absence of tetracycline when inducing HBV replication. HepG2-hNTCP-C4 cells were cultured as described previously^18^.

### Reagents

The compounds used in this study were purchased from Sigma-Aldrich. Entecavir was obtained from Santa Cruz Biotechnology.

### Establishment of subclones of HepAD38 and HepG2.2.15 cells

Limiting dilution was conducted with HepAD38 or HepG2.2.15 cells in a 96 well plate. We over-diluted and seeded the cells with calculating in which 0.1 cell in number was seeded in one well. The medium was changed every three days. After approximately one month, formed cell colonies were transferred to a new 96 well plate and were expanded accordingly to larger size plates. Cells that grew continuously and could repeat culture through passing a freezing stock in Cell Banker (TaKaRa) were named Hep38.2-Tet, Hep38.3-Tet, and Hep38.7-Tet cells (HepAD38 subclones), and HepG2.2.15.7 cells (a HepG2.2.15 subclone).

### Detection of HBs

HBs quantification by ELISA was conducted essentially as described previously^15^ using immunoplates coated with anti-HBs antibody at 1:4,000 dilution. HBs was visualized with horseradish peroxidase-labeled anti-HBs antibody followed by treatment with peroxidase assay kit (Sumitomo bakelite).

### Real time PCR for quantification of HBV DNA and cccDNA

Real time PCR for quantification of HBV DNA and cccDNA were performed essentially as described^15^. The primers and probes used in this study to quantify HBV DNA are 5′-ACTCACCAACCTCTTGTCCT-3′, 5′-GACAAACGGGCAACATACCT-3′, and 5′FAM-TATCGTTGGATGTGTCTGCGGCGT-TAMRA3′ for HBV DNA genotype C, 5′-ACTCACCAACCTCCTGTCCT-3′, 5′-GACAAACGGGCAACATACCT-3′, and 5′FAM-TATCGCTGGATGTGTCTGCGGCGT-TAMRA3′ for HBV DNA genotype A, 5′-AAGGTAGGAGCTGGAGCATTCG-3′, 5′-AGGCGGATTTGCTGGCAAAG-3′, and 5′FAM-AGCCCTCAGGCTCAGGGCATAC-TAMRA3′ for HBV DNA genotype D, and 5′-CGTCTGTGCCTTCTCATCTGC-3′, 5′-GCACAGCTTGGAGGCTTGAA-3′, and 5′FAM-CTGTAGGCATAAATTGGT-TAMRA3′ for HBV cccDNA, respectively.

### Southern blot analysis

The cells were lysed in the buffer [100 mM Tri-HCl (pH8.0), 0.2% NP-40, 150 mM NaCl and protease inhibitor (Complete EDTA-free, Roche]) and free nucleic acids were digested with DNase I and RNase A, followed by treatment with proteinase K to recover nucleocapsid-associated HBV DNA. Detection of nucleocapsid-associated HBV DNA was performed as described previously^37, 38^.

### Cell viability assay and caspase assay

Cell viability was determined by MTT assay and trypan blue staining performed as described previously^39^. Caspase 3/7 activity was evaluated using Caspase-Glo 3/7 assay kit (Promega) according to the manufacturer’s protocol.

### Transient transfection of HBV DNA

HepG2 cells were transfected with a plasmid encoding 1.24 copy of HBV DNA (HBV/Aeus, HBV/C-AT, and HBV/D-IND60)^13^ using TransIT LT1 regent (Mirus) according to the manufacturer’s protocol.

### HBV preparation and infection

HBV preparation and infection were performed as described previously^40^. HBV was recovered from the supernatant of HBV-producing cells including Hep38.7-Tet cells and was concentrated to approximately 200 fold by polyethylene glycol (PEG) precipitation. In the infection assay, HBV was inoculated with 4% PEG8000 for 16 h as described^18, 40^.

### Indirect immunofluorescence analysis

Immunofluorescence was conducted essentially as described^15^. After fixation with 4% paraformaldehyde and permeabilization with 0.3% Triton X-100, the cells were treated with the primary antibodies against HBc (Thermo Fisher Scientific) and tubulin (sigma), and then with Alexa488- or Alexa555-conjugated secondary antibody together with DAPI.

### Coimmunoprecipitation Assay

The cells were lysed with the buffer [100 mM Tris-HCl (pH 8.0), 150 mM NaCl, 0.2% NP-40 and complete protease inhibitor], followed by an immunoprecipitation with anti-FLAG (sigma), anti-tubulin (sigma), anti-HBc antibody (DAKO) or mouse normal IgG as a negative control, essentially as described previously^41^.

### Reporter Assay

Reporter Assay was performed as described^15^.

### Particle gel assay

The cells were lysed in 10 mM Tris–HCl (pH 7.5), 1 mM EDTA, 100 mM NaCl, 0.1% NP-40 and protease inhibitor (Complete EDTA-free, Roche). The lysates were applied to 1% native agarose gel electrophoresis. Detection of capsid was performed essentially as described^42–44^.

### Chemical screening

At three days after seeding, Hep38.7-Tet cells were treated with compounds in the absence of tetracycline. Medium was changed every three days to a fresh medium supplemented with the compounds. At six days posttreatment with compound, culture supernatant was recovered to extract DNA with SideStep lysis and stabilization buffer (Stratagene), and HBV DNA was quantified by real time PCR. Cell viability was simultaneously determined by MTT assay. Compounds reducing the cell viability to less than 80% were eliminated from further evaluation. Normalized HBV DNA levels were calculated with HBV DNA divided by cell viability for each compound.

### Statistics

Statistical significant was determined by using student’s test (*P < 0.05, **P < 0.01, N.S.; not significant).

## Electronic supplementary material

Supplementary Information
