# Supplementary material for: Functional association of cellular microtubules with viral capsid assembly supports efficient hepatitis B virus replication
Source: Sci Rep. 2017 Sep 6;7:10620. doi: 10.1038/s41598-017-11015-4 (PMC5587681; doi:10.1038/s41598-017-11015-4)
Supplement: Supplementary file 1 — Supplementary Information [file 41598_2017_11015_MOESM1_ESM.pdf]

Supplementary Information

Functional association of cellular microtubules with viral capsid assembly supports efficient hepatitis B virus replication

Masashi Iwamoto[1,2], Dawei Cai[3], Masaya Sugiyama[4], Ryosuke Suzuki[1], Hideki Aizaki[1], Akihide Ryo[5], Naoko Ohtani[2], Yasuhito Tanaka[6], Masashi Mizokami[4], Takaji Wakita[1], Haitao Guo[3], Koichi Watashi[1,2,7]*

[1]Department of Virology II, National Institute of Infectious Diseases, Tokyo, 162-8640, Japan, [2]Department of Applied Biological Sciences, Tokyo University of Science, Noda, 278-8510, Japan, [3]Department of Microbiology and Immunology, Indiana University School of Medicine, Indianapolis, 46202, USA, [4]Genome Medical Sciences Project, National Center for Global Health and Medicine, Ichikawa, 272-8516, Japan, [5]Department of Microbiology, Yokohama City University School of Medicine, Yokohama, 236-0004, Japan, [6]Department of Virology and Liver Unit, Nagoya City University Graduate School of Medicinal Sciences, Nagoya, 467-8601, Japan, [7]CREST, JST, Saitama, 332-0012, Japan

* **Address correspondence to:** Koichi Watashi, Ph.D.
Department of Virology II, National Institute of Infectious Diseases, Tokyo, Japan
1-23-1 Toyama, Shinjuku-ku, Tokyo, 162-8640, Japan
Tel: +81-3-5285-1111; Fax: +81-3-5285-1161
E-mail: kwatashi@nih.go.jp

Fig. S1.

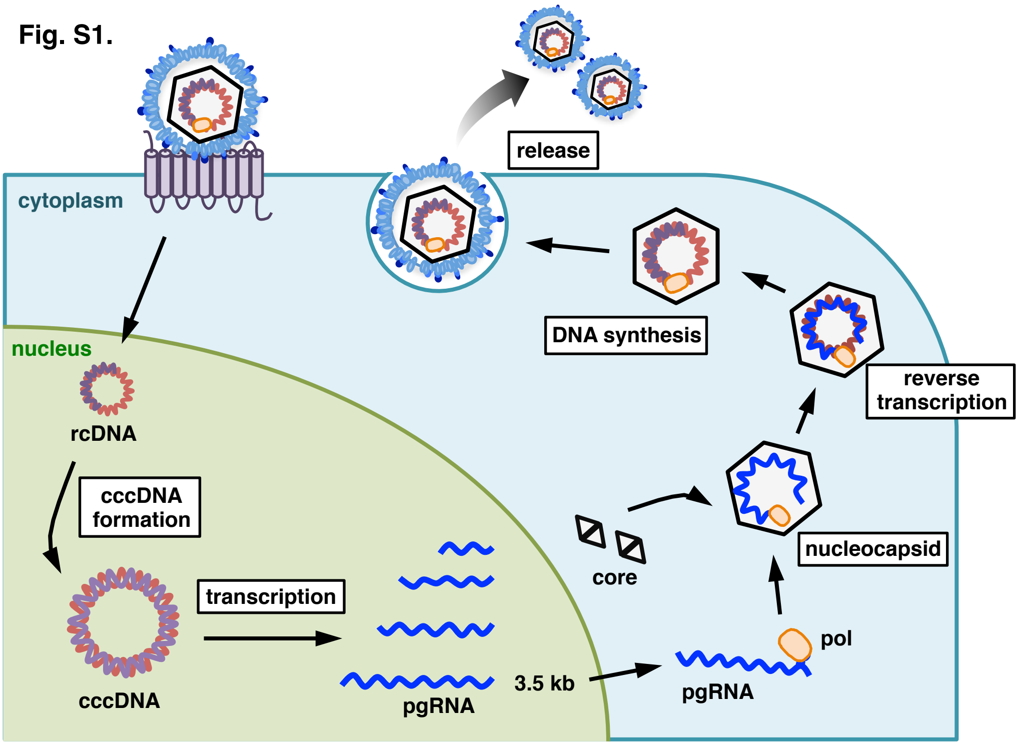

Fig. S1. HBV life cycle. HBV infects into hepatocytes, and then the genome is translocated into the nucleus to form covalently closed circular DNA (cccDNA). One of viral transcripts with approximately 3.5 kb, called pre-genomic RNA (pgRNA), produces polymerase and core protein. pgRNA-polymerase riboprotein complex assembles with core proteins to form nucleocapsid. Through reverse transcription and DNA synthesis inside the nucleocapsid, HBV acquires the envelope and released outside of the cells.

Fig. S2.

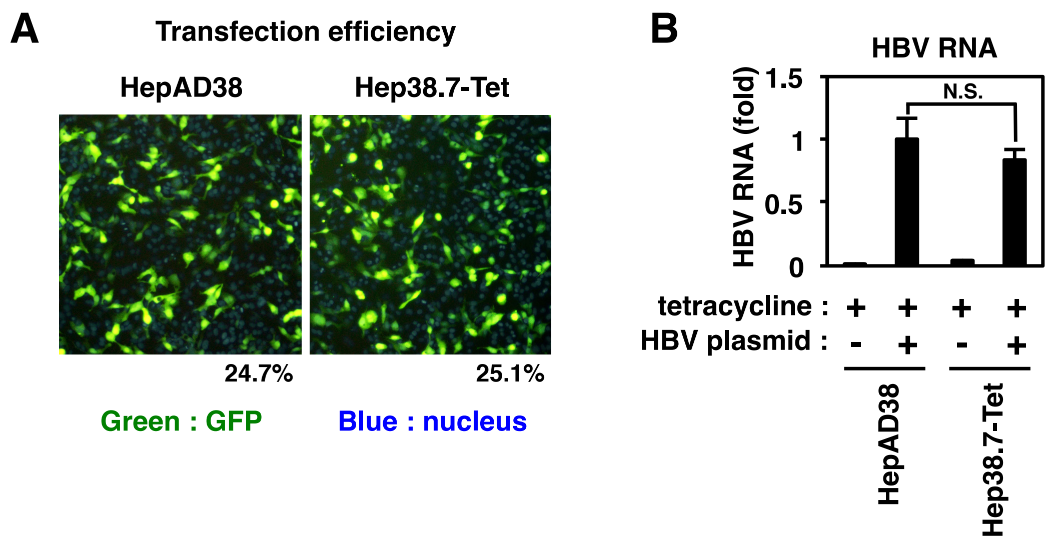

Fig. S2. Transfection efficiency (A) and HBV RNA production (B) in HepAD38 and Hep38.7-Tet cells. (A) HepAD38 and Hep38.7-Tet cells were transfected with a GFP expression plasmid. At 48 h post-transfection, GFP signal was detected by fluorescent microscopy and the percentage of GFP-positive cells was calculated. Transfection efficiency of HepAD38 and Hep38.7-Tet cells was similar. (B) HepAD38 and Hep38.7-Tet cells transfected with an empty vector or an HBV-encoding plasmid were recovered to quantify total HBV RNA. Statistical significance was determined by using Student's t test (*$P < 0.05$, **$P < 0.01$, N.S.; not significant).

Fig. S3.

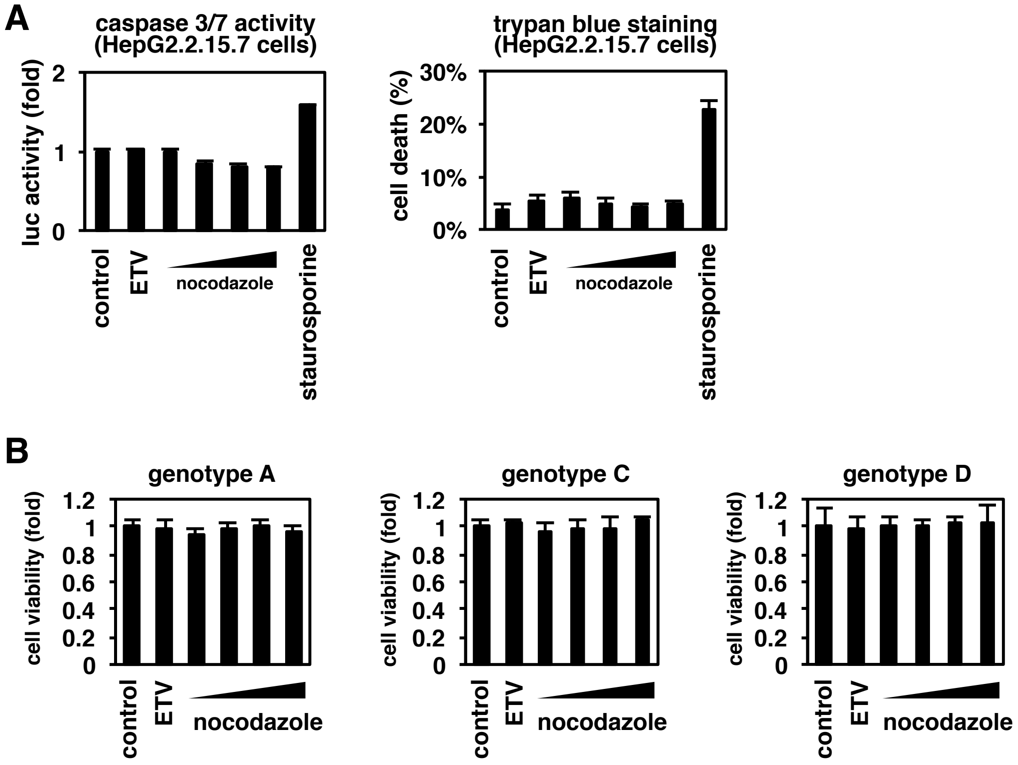

Fig. S3. Cell viability of HBV-replicating cells. (A) HepG2.2.15.7 cells were treated with 1 μM ETV, 0.5, 1, 2.5, and 5 μM nocodazole for 6 days, or with 1 μM staurosporin for 24 h as a positive control to show apoptosis, or left untreated. Cell viability and caspase3/7 activity were quantified by trypan blue staining (right) and caspase assay (left), respectively. (B) Cell viability of HepG2 cells treated with or without 1 μM ETV or 1, 2.5, 5 and 10 μM nocodazole for 72 h after transfection of plasmid encoding different HBV genotypes (A, C, or D) were evaluated by MTT assay.

Fig. S4.

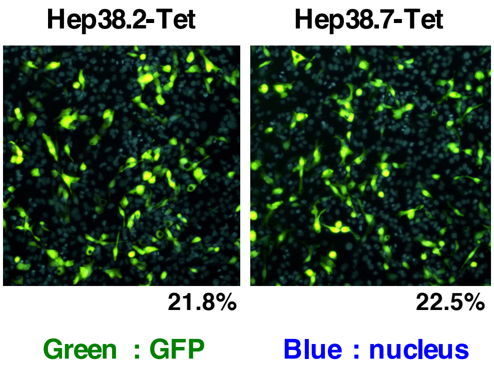

Fig. S4. Transfection efficiency in Hep38.2-Tet and Hep38.7-Tet cells. GFP signal in Hep38.2-Tet and Hep38.7-Tet cells transiently transfected with a GFP expression plasmid was observed by fluorescent microscopy, and the percentage of GFP-positive cells was calculated. No apparent difference in transfection efficiency was observed between Hep38.2-Tet and Hep38.7-Tet cells.
